# A machine learning approach for predicting methionine oxidation sites

**DOI:** 10.1186/s12859-017-1848-9

**Published:** 2017-09-29

**Authors:** Juan C. Aledo, Francisco R. Cantón, Francisco J. Veredas

**Affiliations:** 1 Departamento de Biología Molecular y Bioquímica, Facultad de Ciencias, Universidad de Málaga, Bulevar de Louis Pasteur s/n, Málaga, 29071 Spain; 2 Departamento de Lenguajes y Ciencias de la Computación, Universidad de Málaga, Bulevar de Louis Pasteur s/n, Málaga, 29071 Spain

**Keywords:** Methionine sufoxide, Machine learning, Oxidation prediction, Post-translation modification

## Abstract

**Background:**

The oxidation of protein-bound methionine to form methionine sulfoxide, has traditionally been regarded as an oxidative damage. However, recent evidences support the view of this reversible reaction as a regulatory post-translational modification. The perception that methionine sulfoxidation may provide a mechanism to the redox regulation of a wide range of cellular processes, has stimulated some proteomic studies. However, these experimental approaches are expensive and time-consuming. Therefore, computational methods designed to predict methionine oxidation sites are an attractive alternative. As a first approach to this matter, we have developed models based on random forests, support vector machines and neural networks, aimed at accurate prediction of sites of methionine oxidation.

**Results:**

Starting from published proteomic data regarding oxidized methionines, we created a hand-curated dataset formed by 113 unique polypeptides of known structure, containing 975 methionyl residues, 122 of which were oxidation-prone (positive dataset) and 853 were oxidation-resistant (negative dataset). We use a machine learning approach to generate predictive models from these datasets. Among the multiple features used in the classification task, some of them contributed substantially to the performance of the predictive models. Thus, (i) the solvent accessible area of the methionine residue, (ii) the number of residues between the analyzed methionine and the next methionine found towards the N-terminus and (iii) the spatial distance between the atom of sulfur from the analyzed methionine and the closest aromatic residue, were among the most relevant features. Compared to the other classifiers we also evaluated, random forests provided the best performance, with accuracy, sensitivity and specificity of 0.7468±0.0567, 0.6817±0.0982 and 0.7557±0.0721, respectively (mean ± standard deviation).

**Conclusions:**

We present the first predictive models aimed to computationally detect methionine sites that may become oxidized in vivo in response to oxidative signals. These models provide insights into the structural context in which a methionine residue become either oxidation-resistant or oxidation-prone. Furthermore, these models should be useful in prioritizing methinonyl residues for further studies to determine their potential as regulatory post-translational modification sites.

## Background

Reactive oxygen species (ROS) are well known for their harmful effect on cellular constituents [1]. However, a more nuanced view has emerged during the last years. It is now clear that certain ROS, including H_2_O_2_, can function as messengers [2]. To act as an effective messenger, hydrogen peroxide has to bring about a reversible change in the activity of a protein through post-translational modification (PTM). The amino acids that are used as PTM sites often have a functional group that is able to act as a nucleophile during the modification reaction. In this regard, the sulfur contained in the side chain of cysteine and methionine is liable to be oxidized by H_2_O_2_. Under mild oxidative conditions, cysteine forms cystine through a disulfide bridge, while methionine is preferentially oxidized to methionine sulfoxide. Both oxidation reactions can be reverted through reduction reactions catalyzed by enzymes. Disulfides are reduced back to the thiol form by various reductases [3]. On the other hand, MetO is reduced back to methionine by the enzyme methionine sulfoxide reductase (Msr), present in most aerobic cells [4].

Like phosphorylation of serine, sulfoxidation of methionine is a reversible covalent modification capable of modifying the physicochemical properties of the complete protein, which, in turn, can affect the stability and/or activity of the target protein [5, 6]. Indeed, it has been demonstrated that sulfoxidation of specific methionine residues can lead to both activation [7–9] and inactivation [10, 11] of the modified protein. Moreover, the oxidation of specific methionine sites may also impact the function of a protein in an indirect manner, by facilitating or hindering the occurrence of other functional PTM such as phosphorylation of nearby serine residues [12–14].

The perception that methionine sulfoxidation may provide a mechanism to the redox regulation of a wide range of cellular processes, has stimulated some proteomic studies [15–17]. This proteomic approach, despite the technical difficulties involved in the discrimination between physiological and artifactual modifications, has allowed to identify a considerable number of cellular proteins as possible targets of oxidative signals. Furthermore, these proteomic efforts have allowed to pinpoint the sites of oxidation over the target proteins. Nevertheless, these experimental approaches, besides being expensive, are labor-intensive and time-consuming. In view of this, it is highly desirable to develop *in silico* methods aimed to predict methionine oxidation sites. Indeed, in the field of protein phosphorylation, the prediction of phosphorylation sites using computational tools has attracted considerable research attention [18–20]. Unfortunately, computational approaches to predict methionine oxidation sites have garnered much less attention, and only very recently some efforts have been devoted to this purpose [21].

Herein, we describe predictive models based on computational intelligence, aimed at accurate prediction of methionine sulfoxidation sites.

## Results

For each methionine residue from the training dataset, a total of 76 characteristics were evaluated as described in the “Methods” section. 52 of these characteristics were derived from the primary structure while the remaining 24 characteristics were related to the tertiary structure. These collections of features will be referred to as, *Whole*, *Primary* and *Tertiary*, respectively. Using these different sets of characteristics, we designed a number of machine learning (ML) predictive models, namely random forests (RF) [22], support vector machines (SVM) [23] and neural networks (NN) [24], which were intensively tested in a comparative approach. The results obtained from these comparative studies are presented in the following subsections.

### Predicting methionine oxidation with random forest

The performance of various RF-based models was evaluated in terms of the area under the ROC curve (AUC), accuracy, sensitivity, specificity, F-measure and MCC (Matthews Correlation Coefficient). The results obtained using different subsets of characteristics, for both training and testing datasets, are shown in Table 1 (first four rows of “TRAINING SET” and “TESTING SET” sub-tables from Table 1). In addition to the above described subsets of characteristics, we also used a subset formed by the most relevant features (see “Methods” section). To this end, the characteristics were ranked using the maximum relevance minimum redundancy (mRMR) method [25], which uses a ranking criterion based on the trade-off between the relevance to the output (oxidable) and the redundancy between the input characteristics. In this way, a final subset of 54 features was identified as the optimal (giving the maximum AUC) feature set (see “Methods” section for details).

**Table 1:** Performance rates with three different ML models

Feature set	AUC	Accuracy	Sensitivity	Specificity	F-measure	MCC
TRAINING SET						
RF						
Primary (52)	1.0000	0.8233	1.0000	0.7980	0.5868	0.5756
Tertiary (24)	0.9958	0.7222	1.0000	0.6823	0.4746	0.4607
Whole (76)	1.0000	0.8476	1.0000	0.8258	0.6222	0.6107
mRMR (54)	1.0000	0.8348	1.0000	0.8111	0.6031	0.5918
SVM						
Primary (52)	1.0000	0.4955	1.0000	0.4231	0.3322	0.2903
Tertiary (24)	0.9403	0.9232	0.8571	0.9327	0.7368	0.7024
Whole (76)	0.9927	0.9910	0.9592	0.9956	0.9641	0.9590
mRMR (54)	0.9952	0.9821	0.9490	0.9868	0.9300	0.9200
NN						
Primary (52)	0.7148	0.6492	0.6020	0.6559	0.3010	0.1764
Tertiary (24)	0.7981	0.7273	0.7143	0.7291	0.3966	0.3132
Whole (76)	0.7827	0.6402	0.8061	0.6164	0.3599	0.2822
mRMR (54)	0.7933	0.6786	0.8061	0.6603	0.3863	0.3156
TESTING SET						
RF						
Primary (52)	0.7002	0.5969	0.8125	0.5664	0.3333	0.2500
Tertiary (24)	0.8014	0.6357	0.8750	0.6018	0.3733	0.3155
Whole (76)	0.8413	0.7597	0.8125	0.7522	0.4561	0.3998
mRMR (54)	0.8462	0.7597	0.7500	0.7611	0.4364	0.3668
SVM						
Primary (52)	0.5603	0.4264	0.7500	0.3805	0.2449	0.0894
Tertiary (24)	0.4701	0.2791	0.6250	0.2301	0.1770	-0.1106
Whole (76)	0.6831	0.7984	0.4375	0.8496	0.3500	0.2431
mRMR (54)	0.7406	0.7907	0.4375	0.8407	0.3415	0.2320
NN						
Primary (52)	0.5669	0.5504	0.4375	0.5664	0.1944	0.0026
Tertiary (24)	0.8291	0.7364	0.8125	0.7257	0.4333	0.3742
Whole (76)	0.7959	0.6589	0.7500	0.6460	0.3529	0.2661
mRMR (54)	0.8208	0.7132	0.8750	0.6903	0.4308	0.3839

### Comparison with other machine learning models

To account for the potential of RF as an effective ML approach to predict the oxidation of methionine, we have compared it with two other classical ML models: SVM and NN (see “Methods” section). The performance of these alternative methods is also summarized in Table 1. These results showed differences in favor of RF, with respect to SVM and NN, as RF gave high AUC and accuracy rates with a better balance between sensitivity and specificity rates for data from the testing set.

**Fig. 1:**
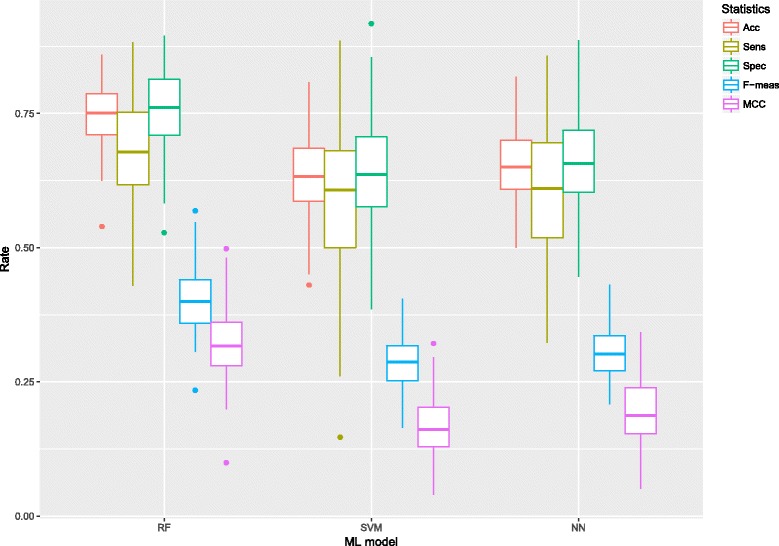
Performance rates distributions for bootstrapping resamples. Box-plots of the performance rates on the testing sets (after ROC’s cut-off probability adjustment on the evaluation sets) for bootstrapping resamples. Data set *mRMR 54 features*. Number of resamples = 100

However, as those results in Table 1 correspond to single ML models applied on a same training/testing set, a more comprehensive evaluation of each ML-model’s predictive potential was needed. In this vein, Table 2 and Fig. 1 show the results from a bootstrapping strategy: for each ML model and feature subset (*Primary*, *Tertiary*, *Whole* and *mRMR*), 100 bootstrap re-samples were generated and 10-fold cross-validation (with 5 repetitions) were used to train and fit each model. Mean performance rates and standard deviation on the training and testing sets (after ROC’s cut-off probability adjusting on the evaluation sets) are shown in Table 2. The best overall results on the testing sets (high accuracy rate with balanced sensitivity and specificity) were obtained with RFs, showing significant differences with respect to SVMs and NNs (see t-test *p*-values in Table 3). Remarkably, very similar results were obtained with both the *mRMR* subset and the whole set of 76 characteristics. In general, SVMs and NNs showed similar efficacy rates, with accuracy numbers that were lower than those given by the RFs and worse balances between sensitivity specificity rates (see Table 3 and Fig. 1).

**Table 2:** Performance rates for three different ML approaches: mean (sd)

Feature set	AUC	Accuracy	Sensitivity	Specificity	F-measure	MCC
TRAINING SET						
RF						
Primary	1.0000 (0)	0.8957 (0.0480)	1 (0)	0.8807 (0.0546)	0.7176 (0.0938)	0.7054 (0.0920)
Tertiary	0.9996 (0.0003)	0.8316 (0.0591)	1 (0)	0.8074 (0.0674)	0.6096 (0.0898)	0.5977 (0.0882)
Whole	1.0000 (0)	0.8948 (0.0533)	1 (0)	0.8797 (0.0609)	0.7192 (0.1053)	0.7071 (0.1046)
mRMR	1.0000 (0)	0.8932 (0.0480)	1 (0)	0.8777 (0.0550)	0.7138 (0.0966)	0.7015 (0.0960)
SVM						
Primary	0.9997 (0.0011)	0.9069 (0.1990)	0.9990 (0.0034)	0.8939 (0.2270)	0.8751 (0.2584)	0.8670 (0.2747)
Tertiary	0.9924 (0.0090)	0.7425 (0.1501)	0.9865 (0.0217)	0.7077 (0.1729)	0.5562 (0.2335)	0.5390 (0.2407)
Whole	0.9992 (0.0025)	0.9310 (0.1542)	0.9980 (0.0058)	0.9210 (0.1772)	0.8936 (0.2254)	0.8874 (0.2370)
mRMR	0.9995 (0.0018)	0.9044 (0.1766)	0.9982 (0.0043)	0.8907 (0.2026)	0.8545 (0.2561)	0.8463 (0.2690)
NN						
Primary	0.9482 (0.0339)	0.7248 (0.1607)	0.9377 (0.0416)	0.6938 (0.1841)	0.5195 (0.1975)	0.4835 (0.2133)
Tertiary	0.9336 (0.0227)	0.7552 (0.1040)	0.9079 (0.0322)	0.7334 (0.1195)	0.5082 (0.1322)	0.4706 (0.1378)
Whole	0.9616 (0.0247)	0.8273 (0.1170)	0.9491 (0.0327)	0.8098 (0.1333)	0.6292 (0.1883)	0.6063 (0.1958)
mRMR	0.9533 (0.0232)	0.7897 (0.1160)	0.9373 (0.0314)	0.7684 (0.1325)	0.5696 (0.1738)	0.5413 (0.1822)
TESTING SET						
RF						
Primary	0.6947 (0.0416)	0.6207 (0.0666)	0.6737 (0.1296)	0.6139 (0.0883)	0.3026 (0.0439)	0.1936 (0.0573)
Tertiary	0.7614 (0.0375)	0.6975 (0.0485)	0.7064 (0.1029)	0.6959 (0.0633)	0.3638 (0.0463)	0.2781 (0.0547)
Whole	0.7957 (0.0355)	0.7458 (0.0622)	0.6849 (0.1195)	0.7540 (0.0813)	0.4003 (0.0563)	0.3205 (0.0625)
mRMR	0.7998 (0.0334)	0.7468 (0.0567)	0.6817 (0.0982)	0.7557 (0.0721)	0.4003 (0.0562)	0.3190 (0.0622)
SVM						
Primary	0.5660 (0.0431)	0.5604 (0.0847)	0.5383 (0.1381)	0.5641 (0.1112)	0.2286 (0.0414)	0.0688 (0.0573)
Tertiary	0.6480 (0.0534)	0.6434 (0.0825)	0.5500 (0.1329)	0.6561 (0.1070)	0.2741 (0.0459)	0.1437 (0.0605)
Whole	0.6753 (0.0424)	0.6441 (0.0704)	0.6037 (0.1301)	0.6501 (0.0954)	0.2924 (0.0417)	0.1744 (0.0498)
mRMR	0.6700 (0.0450)	0.6348 (0.0802)	0.5986 (0.1309)	0.6398 (0.1047)	0.2865 (0.0461)	0.1641 (0.0585)
NN						
Primary	0.5601 (0.0479)	0.5477 (0.0907)	0.5465 (0.1349)	0.5474 (0.1178)	0.2274 (0.0411)	0.0637 (0.0567)
Tertiary	0.6887 (0.0470)	0.6662 (0.0687)	0.5998 (0.1412)	0.6745 (0.0907)	0.3047 (0.0523)	0.1907 (0.0658)
Whole	0.6846 (0.0469)	0.6650 (0.0680)	0.5793 (0.1194)	0.6765 (0.0886)	0.2981 (0.0453)	0.1791 (0.0581)
mRMR	0.6903 (0.0486)	0.6573 (0.0696)	0.6101 (0.1224)	0.6640 (0.0903)	0.3044 (0.0474)	0.1900 (0.0627)

**Table 3:** Models comparison. T-test *p*-value from bootstrap results on the testing sets

Feature set	RF-SVM	RF-NN	SVM-NN
AUC			
Primary	1.337807e-53	1.656090e-52	3.629288e-01
Tertiary	7.466593e-08	7.749183e-10	3.076722e-01
Whole	1.620777e-11	1.207725e-10	6.687422e-01
mRMR	5.736385e-04	1.122952e-05	3.027066e-01
Accuracy			
Primary	7.466593e-08	7.749183e-10	3.076722e-01
Tertiary	1.620777e-11	1.207725e-10	6.687422e-01
Whole	5.736385e-04	1.122952e-05	3.027066e-01
mRMR	1.110837e-35	7.810002e-38	5.302538e-01
Sensitivity			
Primary	4.838807e-26	9.923600e-27	8.419212e-01
Tertiary	7.067182e-08	2.630463e-04	3.507737e-02
Whole	3.771161e-17	6.241079e-09	1.096249e-02
mRMR	1.650447e-03	5.410619e-02	1.924156e-01
Specificity			
Primary	7.035627e-39	8.036713e-20	3.721847e-07
Tertiary	1.059365e-30	6.066923e-15	1.807435e-05
Whole	1.072350e-21	9.069624e-16	3.319756e-02
mRMR	7.569818e-06	2.475176e-09	1.699726e-01
F-measure			
Primary	1.900064e-14	8.911586e-10	4.341598e-02
Tertiary	1.385330e-43	3.632520e-39	5.440616e-01
Whole	8.253875e-35	1.802488e-31	3.612268e-01
mRMR	5.984561e-23	4.366711e-19	3.520361e-02
MCC			
Primary	9.137039e-07	8.985178e-06	5.212807e-01
Tertiary	1.701737e-16	1.821765e-13	8.146449e-02
Whole	6.201029e-44	2.659090e-33	2.914253e-03
mRMR	4.996287e-36	3.033082e-28	7.392408e-03

**Fig. 2:**
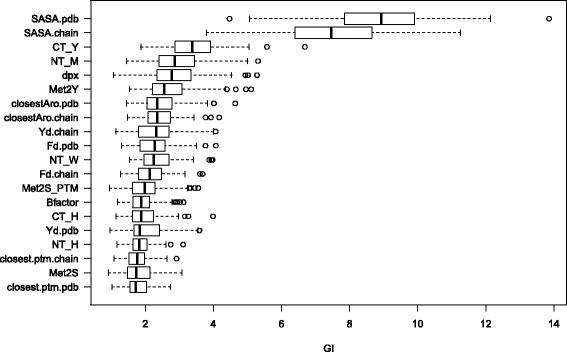
Variable Importance. Box-plots of the GI of the 20 most relevant predictors for the RF classifier. From top to bottom: variables in decreasing order of average GI (100 bootstrapping resamples). Dataset: *mRMR 54 features*

The quantification of the predictive importance of each variable is a key factor to interpret data and to understand the phenomena underlying methionine oxidation. Thus, we resorted to the Gini-index importance to assess the relevance of the variable used for the RF classifiers as input characteristic. Fig. 2 shows the 20 most relevant variables as estimated by the RF on the training set (100 bootstrap resampling), along with the distribution (box-plot) of their averaged decrease in Gini-index (see “Methods” section). As it can be observed, the accessibility to the solvent, the proximity to other methionyl residues and the distance to the closest aromatic residue are among the variables with the highest predictive importance (Fig. 2).

## Discussion

Protein-bound methionine is readily oxidized to methionine sulfoxide, which can drastically affect the biological activity of the modified proteins. Although this fact has been known for many years now, our perception of the functional implication of methionine sulfoxidation has evolved over time. Initially, this chemical modification was detected in proteins that had been purified from tissues following laborious experimental procedures. Hence, there was a reasonable doubt of whether the observed modification was present in the natural tissues, or whether it arose from some oxidation during the manipulations in vitro [26]. A decade later, it was clear that the oxidation of methionine in proteins takes place in vivo [27]. However, the presence of methionine sulfoxide in proteins was considered just as an inevitable and harmful consequence of oxidative stress. Later on, the regard of methionine oxidation as mere oxidative damage would give pass to a more benign judgment.

The finding that oxidation of protein-bond methionine residues to methionine sulfoxide is one of the few protein oxidation events that are reversible in vivo, led to the appealing hypothesis of methionine residues as endogenous antioxidants in proteins [28]. Indeed, reversible oxidation/reduction of methionine residues in proteins can serve as a scavenger system to remove ROS, and the importance of methionine oxidation in the antioxidation defense has gathered strong experimental evidences since then [29, 30]. On the other hand, although ROS have traditionally been thought as harmful by-products of respiratory metabolism, that notion has slowly given way to a more nuanced view of ROS as important signaling molecules [1]. In this context, a new functional role for methionine modification can be envisioned. Methionines that undergo sulfoxidation may serve as PTM sites fulfilling a signaling role, acting as on/off sensors of oxidative stress in certain proteins. A number of such proteins has already been identified [31–34].

Our current awareness of the functional relevance of methionine oxidation at certain sites, demands tools for the prediction of such sites. As a first step towards this goal, in this study we have developed machine learning models for predicting whether a given methionine residue would be oxidized in vivo after an oxidative challenge. In the past, driven by the interest to expand the shelf life of therapeutic proteins, considerable effort has been devoted to predict the reactivity in vitro of methionine residues towards oxidants, using for this purpose molecular modeling [35]. However, because of the limited number of proteins analyzed and the nature of the data used (obtained from in vitro kinetic assays) the use of these molecular models cannot be extrapolated to a more general framework of methionine oxidation prediction. In contrast, herein we have used a large collection of data encompassing over hundred proteins containing 122 methionyl residues that have been empirically detected as methionine sulfoxide. The fact that these sulfoxidized methionines are present within the cells, means that the proteome data used in the current study represents a steady-state situation, in which oxidation after hydrogen peroxide challenge is balanced by reduction catalyzed by methionine sulfoxide reductases. Therefore, our study is, to the best of our knowledge, the first attempt to train and test computational models aimed to predict the oxidation status of protein-bound methionines, when such protein are found into their subcellular environment.

In this work, we have used machine learning models to predict the oxidation of methionine in protein sequences. To this end, all the models we have been dealing with handled two output classes: modified and unmodified methionine sites, where the negative category (non-oxidized methionine) is defined by the absence of the modification. It may be possible that some of the methionine sites labeled as negative would be actually modified sites, but the experimental procedure failed to detect them? Although such a possibility never can be fully ruled out, it seems unlikely. Indeed, protein abundance is a major factor for the detection of PTMs by mass spectrometry. To this respect, an important characteristic of our ML approach is that each methionine site belonging to the negative dataset had its own internal control. Since negative methionines were obtained from proteins containing at least one positive methionine, we can be confident that the non-oxidized methionine was present at equimolar concentration with respect to other methionine detected as MetO during the same experiment. Nevertheless, a caveat that should be taken into consideration is that the whole dataset come from a single proteomic study using Jurkat cells [15]. Whether the cellular processes taken place in this cell line represent those operating in animal tissues, is an issue that remains to be solved [36]. In any event, future effort directed to identify new methionine sulfoxidation sites in vivo, using different species, tissues and experimental conditions, will lead to improved predictive models.

The unbalanced distribution of the output classes (oxidized vs non-oxidized) and the proportion of missing data in the dataset are two characteristics that deserve discussion because they affect the performance of the predictive models. The former has to do with the severe class imbalance (the positive dataset only represents 12.5*%* of the whole dataset). When training and tuning the predictive models, we had to deal with this unwanted issue. Fortunately, the unbalanced distribution problem could be resolved using sampling techniques or ROC curve post-processing approaches. On the other hand, missing data can dramatically affect the effectiveness of the classifiers if not appropriately treated. Moreover, the predictive models used in the current study cannot deal with missing values, which make missing data imputation unavoidable. Three different missing data imputation methods have been tested in our study, k-nearest neighbors (KNN) imputation, median imputation and bagging imputation [37]. KNN imputation was carried out by finding the *k* closest samples (Euclidean distance) in the training set. Imputation via medians takes the median of each predictor in the training set and used them to fill missing values. This method is simple and fast, but treats each predictor independently and may be inaccurate. Imputation via bagging fits a bagged tree model for each predictor (as a function of all the other features). This method, which is simple and accurate, gave us the best results in our study although it had higher computational cost.

Since protein sequences are easily determined and easy to work with, initially we resorted to features that could be extracted using only protein sequence information, to build the so-called *Primary* models. Despite the limitation of disregard valuable 3D structural information, these models performed modestly well (Table 2), with balanced sensitivity and specificity in spite of a remarkable imbalance between the total numbers of oxidized and non-oxidized methionine sites in the training samples. Nevertheless, when features related to the spatial structure of the protein were included into the models, their performance improved substantially. This finding is consistent with previous studies demonstrating the importance of structural variables (such as the solvent accessible area of the methionine and its spatial proximity to aromatic residues) in determining the oxidation state of methionyl residues in the proteins within living cells [38]. Interestingly, the use of computational techniques to filter features on the base of their high relevance and low redundancy (mRMR), allowed us to conclude that a reduced number of features (54 out of 76) was enough to obtain the best results.

With respect to the different ML approaches tested herein, the best performance was obtained using RF, while SVM and NN behave worse in general when compared to RF (Table 2 and Fig. 1). There is not a clear reason why this should be that way. However, again the heterogeneous nature of the data, including the intrinsically unbalanced distribution of the output classes, make the RF a better ML approach for this particular problem of methionine oxidation site prediction. The “ensemble nature” of RF (a large pool of decision trees is built during the training phase) does its best to deal with the challenge of predicting new input patterns as those found in the testing sets, thus giving high performance rates while the balance between sensitivity and specificity remains. Nevertheless, since the limitation of available data and the unbalanced characteristic of the dataset may affect the performance of the classifier, further work for refining and improving the prediction model will be carried out using additional classification methods and additional dataset when they become available. We also provide a stand-alone program based on the RF model described herein. This software can be downloaded from google.drive.scripts, where any interested user will also find detailed use instructions.

Phosphorylation is the most common post-translational modification [39]. Many of the cellular responses triggered by oxidative stress are known to be mediated, at some point, by signaling cascades involving protein phosphorylation [40, 41]. Recent studies have suggested that the crosstalk between serine/threonine phosphorylation and methionine sulfoxidation may serve to fine-tune the cellular response to oxidative signals [12, 14]. In line with these previous works, we have observed that including features related to phosphorylation information (see *Methods* for details) in the predictive model of methionine oxidation does contribute to its performance (see the list of relevant features filtered by the mRMR algoritm, as well as Fig. 2). All in all, these works point to a relevant role for methionine oxidation in the regulation of protein function.

## Conclusions

In this study we have designed and tested computational models to predict methionine oxidation sites. High accuracy rates as well as balanced specificity and sensitivity values were obtained. The best performances were obtained when random forests were used, while neural networks and support vector machines behaved less effectively, in general.

From the 76 features used in the design of our predictive models, some variables related to the protein structure, such as solvent accessibility (SASA) and the proximity of aromatic residues, have been identify among those making the highest contribution to the predictive power of the random forest classifier. Some characteristics regarding phosphorylation, such as the distance to the closest phosphorylable residue, have also been detected as relevant features. This fact supports the hypothesis of methionine sulfoxidation playing an important role in the crosstalk with protein phosphorylation.

As our understanding of the role played by methionine sulfoxidation in all aspects of cellular biology continues to expand, these computantional predictive models will become increasingly valuable, especially in hypothesis-driven investigations. Moreover, the availability of reliable predictive tools should stimulate further investigations aimed to gain a better understanding of the interplay between sulfoxidation and phosphorylation during cellular redox signaling.

## Methods

### Datasets

Data regarding methionine residues detected as methionine sulfoxide in vivo were taken from reference [15]. This set was further curated to exclude protein entries that did not contain at least one methionine showing a degree of oxidation, as defined in [15], equal or greater than 20%. Using PDB cross-references from UniProt (http://www.uniprot.org), this collection was further constrained to those proteins with known structure. In general, since many proteins were homooligomers, most crystal structures yielded a large number of duplicated observations, which were searched for and eliminated using a R script. Eventually, after removing redundancy and filtering out low quality structures (for instance, those where the target methionine did not appear resolved), we assembled a collection of 113 unique polypeptides of known structure, containing 975 methyonil residues, 122 of which were oxidation-prone (positive dataset) and 853 were oxidation-resistant (negative dataset).

### Feature extraction

For each methionine residue from the dataset described above, a total number of 76 features were extracted. These features included 20 variables of the type NT_X, defined as the number of positions in the protein sequence from the analysed methionine to the closest X residue toward the N-terminus, where X belong to the set of 20 proteinogenic amino acids. Similarly, other 20 features of the type CT_X were assessed, in this occasion, counting towards the C-terminus.

Four additional features were related to the conservation of the considered methionine during evolution. To assess these features, besides the human sequence, the orthologous proteins from *Pan troglodytes*, *Gorilla gorilla*, *Rattus norvegicus*, *Bos taurus*, *Gallus gallus*, *Xenopus tropicalis* and *Danio rerio* were aligned. These alignments were used to compute the Shannon entropy according to the equation:

$$ entropy = - \sum_{i = 1}^{21} f_{i} log_{21}(f_{i}), $$

where *f*_*i*_ is the relative frequency of the symbol *i* at the analysed position across the alignment. Thus, for instance, *f*_*M*_ stands for the relative frequency of methionine. The logarithmic base was taken 21 because in addition to the 20 proteinogenic amino acids, the symbol ‘-’ was considered when indels were present. For each analysed methionine, the variables mean.entropy and sd.entropy were computed as the mean and standard deviation, respectively, of the entropy determined at all the positions of the corresponding protein.

Eight further features related with PTM sites were evaluated. Concretely, the variables Met2S, Met2T and Met2Y inform about the distance, in the primary structure, between the analysed methionine and the closest serine, threonine and tyrosine phospho-acceptor, respectively. It should be noted that

$$Met2X = \text{min}(NT\_X, CT\_X). $$

On the other hand, Met2S_PTM, Met2T_PTM and Met2Y_PTM collect the distances to the closest corresponding phosphosites. That is, to the closest phospho-acceptor that has been shown to be phosphorylatable [42]. The other two PTM-based features were closer10res, defined as the number of phosphorylatable residues in a radius of 10 amino acids from the analysed methionine, and away.ptm calculated according to the following expression:

$$away.ptm = \underset{X \in \{S, T, Y\}}{\text{min}}(Met2X\_PTM). $$

The 52 features described hitherto can be extracted from the primary structure of the involved proteins. However, to compute the 24 features that we will introduce next, information about the 3D structure of the protein was essential.

Thus, we defined and computed four new variables related to PTM sites. The first of these variables, referred to as closest.ptm.chain, gives the distance in ångströms between the considered methionine and the closest phosphorylatable residue (either Ser, Thr or Tyr experimentally shown to be phosphorylated) present in the same polypeptide chain that the methionyl residue. If we remove the constraint of both sites having to be intrachain, then we will be dealing with the feature closest.ptm.pdb. The feature closer10A.chain provides the number of phosphorylatable sites, found on the same polypeptide chain, within a sphere of radius 10Å centred at the relevant methionine. Analogously, closer10A.pdb gives the number of phosphorylatable sites within the sphere, regardless of the chain hosting them.

In a recent work we reported that methionyl residues forming part of an S-aromatic motif are less prone to be oxidized [38]. Therefore, 16 additional features related to this non covalent bond were used. Concretely, Xd.chain was defined as the distance in ångströms between the sulfur atom from the analysed methionine and the nearest X aromatic residue within the same polypeptide chain, being X either Y (Tyr), F (Phe) or W (Trp). If the aromatic residue is allowed to be in a different polypeptide molecule, we refer to this feature as Xd.pdb. The variables nX.chain and nX.pdb inform about the number of X aromatic residues (within the same polypeptide molecule or not, respectively) at a distance < 7Å from the methionine. The feature numberBonds.chain was computed according to:

$$numberBonds.chain = \sum_{X \in \{Y, F, W\}} nX.chain. $$

Similarly, numberBonds.pdb was defined as:

$$numberBonds.pdb = \sum_{X \in \{Y, F, W\}} nX.pdb. $$

In addition, the variables closestAro.chain and closestAro.pdb were computed as:

$$\begin{array}{*{20}l} closestAro.chain &= \underset{X \in \{Y, F, W\}}{\text{min}} (Xd.chain),\\ closestAro.pdb &= \underset{X \in \{Y, F, W\}}{\text{min}} (Xd.pdb). \end{array} $$

Other two features, SASA.chain and SASA.pdb, were related to the solvent accessible surface area of the methionine residue. These variables were assessed with the program DSSP [43] and either the atomic coordinates of the single polypeptide chain harboring the methionine (for SASA.chain), or the atomic coordinates of the whole protein (for SASA.pdb).

The B factor of the sulfur atom from the methionine of interest extracted from the PDB file used was recorded in the variable Bfactor.

Finally, dpx measures the depth of the sulfur atom from the considered methionine, defined as the distance in ångströms between the S atom and the closest atom from the protein exposed to the solvent [44].

The data file with all these extracted features used in our study is available at github.data

### Machine learning methods

In the current study we used RFs to design predictive models of methionine oxidation sites. RFs are ensemble machine learning methods for classification, that function by constructing a large pool of decision trees during the training phase. The final output will be the mode of the classes given by the individual trees in the pool. The method combines Breiman’s ‘bagging’ idea and the random selection of features (i.e. predictor-set split) in order to construct a collection of decision trees with controlled variation [22].

The quantification of the predictive importance of each variable was carried out by means of the Gini-index Importance (GI). The Gini-index [45] for a given node of a decision tree can be defined as

$$p_{1} (1 - p_{1}) + p_{2} (1 - p_{2}), $$

where *p*_1_ and *p*_2_ are the “class 1” and “class 2” probabilities, respectively. For a binary-classification problem, *p*_1_+*p*_2_=1 and the previous equation could be written as 2*p*_1_ *p*_2_. The Gini-index minimizes when either *p*_1_ or *p*_2_ drives towards zero, and maximizes when *p*_1_=*p*_2_, i.e. when the node is “least pure”. The GI uses the decrease of Gini-index (impurity) after a node split as a measure of variable relevance. The average decrease in Gini-index over all trees in the RF defines the GI.

In general, when it comes to predictive performance, there are cases where SVMs do better than RFs, and vice versa. The same is true for NNs with respect to other ML approaches. Thus, for comparative purposes we also developed classifiers based on SVM [23], as well as on NNs [24].

#### Model tuning

For RF model-fitting in our experiments regarding methionine oxidation, the only sensible tuning hyper-parameter would be the number of variables (predictors) randomly sampled as candidates at each split (usually known as mtry). We fixed the value of this parameter at the optimal recommended value $\lfloor \sqrt {number\ of\ predictors}\rfloor $ [22, 46]. On the other hand, the number of trees to grow was fixed to 1000 to ensure that every input pattern could be predicted at least a few times [47].

For SVMs, a Gaussian radial basis function (RBF) kernel $k(x, x^{\prime }) = e^{-\sigma ||x - x^{\prime }||^{2}}$ was used (being *k* a function that calculates the inner product 〈*Φ*(*x*),*Φ*(*x* ^′^)〉 of two vectors *x*, *x* ^′^ for a given projection *Φ*:*X*→*H*). The problem of model selection (parameter tuning) was partially addressed by an empirical observation for the Gaussian RBF kernel, where the optimal values of the hyper-parameter *σ* are known to lie in between the 0.1 and 0.9 quantile of the ||*x*−*x* ^′^|| statistics [48, 49]. Thus, a sample of the training set was used to estimate these quantiles. Any value of *σ* comprised within the quantile interval results in good performance. In this way, the *σ* parameter was automatically estimated. Additionally, the optimal hyper-parameter *cost*, that represents the cost of constraints violation and stands for the ‘C’-constant of the regularisation term in the Lagrange formulation, was tuned as the one of 12 incremental values in $\{2^{i}\}_{i=-2}^{9}$ that optimises the area under the ROC curve (AUC) of the SVM classifier.

Fully connected single-hidden-layer feed-forward NNs—Multilayer Perceptrons (MLP) [50]—were also constructed and trained with different combinations of parameters to search for the best performance rates in the prediction of methionine oxidation. Optimisation of the NNs was done via the error back-propagation algorithm [50]. The network size (i.e., number of *hidden units* in the single hidden layer) and *weight* decay were the tuned parameters, selecting the combination of values that provided the highest AUC. All the trained MLPs had a number of outputs that was equal to the number of classes (i.e. *n*=2), with logistic activation function for all the hidden and output neurons. Weights were randomly initialised, and maximum number of epochs was fixed to 100 [51].

For each predictive model, the best values for the fitted parameters are computed as those giving the highest averaged AUC via 10-fold cross-validation on the training dataset (in Table 4 the best hyper-parameters for each ML model in Table 1 are shown).

**Table 4:** Model tuning. Best hyper-parameters

Feature set	RF		SVM		NN	
	mtry	Number of trees	Sigma	C	Size	Decay
Primary	7	1000	0.01124415	8	15 0.003162278	
Tertiary	4	1000	0.04226239	8	3	0.0001995262
Whole	8	1000	0.007670497	4	1	0.001584893
mRMR	7	1000	0.01050984	4	19	0.001584893

#### Resampling methods for model fitting

The data set was divided into three independent sets, 80% (98 ‘positive’; 683 ‘control’) patterns for training, 6.7*%* (8 ‘positive’; 57 ‘control’) patterns for evaluation (these pattern set is used to compute the optimal threshold for the ROC curves) and, finally, 13.3*%* (16 ‘positive’; 113 ‘control’) for testing. To preserve the unbalanced nature of the original class distribution within the splits, a stratified random sampling strategy was used. To estimate the efficacy of the prediction model across the training set, six performance measures—AUC, accuracy, sensitivity, specificity, F-measure and Mathews-Correlation-Coefficient (MCC)—of the out-of-bag (OOB) samples for 10-fold cross-validation with 5 repetitions (50 re-samplings) were calculated and the mean and standard deviation of those rates were computed. To compute the latter five performance measures, and given the following general table for any binary classification problem (with two classes: Yes/No),



where *TP*, *FP*, *TN* and *FN* stand for *true positive*, *false positive*, *true negative* and *false negative*, respectively, we have used the following well-known formulae:

- *accuracy* =(*T* *P*+*T* *N*)/(*T* *P*+*T* *N*+*F* *P*+*F* *N*),
- *sensitivity* =*T* *P*/(*T* *P*+*F* *N*),
- *specificity* =*T* *N*/(*T* *N*+*F* *P*),
- *F-measure* $= 2 \frac {precision \times sensitivity}{precision + sensitivity}$, where *p* *r* *e* *c* *i* *s* *i* *o* *n*=*T* *P*/(*T* *P*+*F* *P*),
- *MCC* $= \frac {TP \times TN - FP \times FN}{\sqrt {(TP + FP) (TP + FN) (TN + FP) (TN + FN)}}$.

With respect to the two last performance measures, i.e. the F-measure and MCC, although both of them have been included in our analyses because they both are usually used in machine learning as measures of the quality of binary classifications, the F-measure has to be taken with caution, as it does not take the true negatives into account. For this reason, and given that our dataset is seriously unbalanced towards the negative samples, the MCC may be preferable to assess the performance of our binary classifiers.

The entire training set was used to fit a final model and its performance was finally measured on the testing set. For bootstrap resampling (see “Results” section), 100 random resamples were generated and 10-fold cross-validation (with 5 repetitions) was used to train and fit each model (RF, SVM and NN). The *caret* R package [52, 53] (R version 3.3.3) has been used for model fitting with SVM (package *kernlab* [49]), NN (package *RSNNS* [51]) and RF (package *randomForest* [47]).

One of the more severe circumstances that can dramatically affect the effectiveness of prediction models is class imbalance, i.e. the unbalanced relative frequency of one class in the training set as compared to the other class. In our study, class imbalance is inherent to the procedure being followed for data acquisition (see “Datasets” section): of the complete set of methionine residues found in the 113 polypeptides analysed, only 122 out of 975 appeared as oxidised, i.e. a mere 12.5*%*. This can result in predictive models that can easily get high accuracy rates at the expense of unacceptable sensitivity figures. For example, the most ‘naïve’ predictive model consisting in classifying all methionine residues as ‘non oxidised’ would give 87.5*%* accuracy and 100% specificity, but an unwelcome 0% sensitivity. To further characterize this sensitivity issue, we launched a pool of 1000 “random predictions” over the entire dataset. For each of these random predictions, 12.5*%* of the 975 patterns were randomly chosen as oxidized sites. In this way, the mean accuracy (78.1*%*) and specificity (87.5*%*) were high enough, but, as expected, the mean sensitivity was unacceptably low, 12.4*%* (standard deviation 0.0071, 0.0283 and 0.0040, respectively).

To counteract the negative effects of class imbalance, different approaches have been proposed in the literature [37]. These approaches include model tuning (using metrics alternative to accuracy such as ROC, Cohen’s Kappa or sensitivity), adjusting of prior probabilities, cost-sensitive training, use of alternative ROC-curve cutoffs, or use of specific sampling methods. In the current study a combination of the two latter gave the best results. Prior to model tuning and fitting, we used the down-sampling technique to get a more balanced training dataset. The general idea of this method is to artificially down-sample the majority class (i.e. ‘non oxidised’ class).

**Fig. 3:**
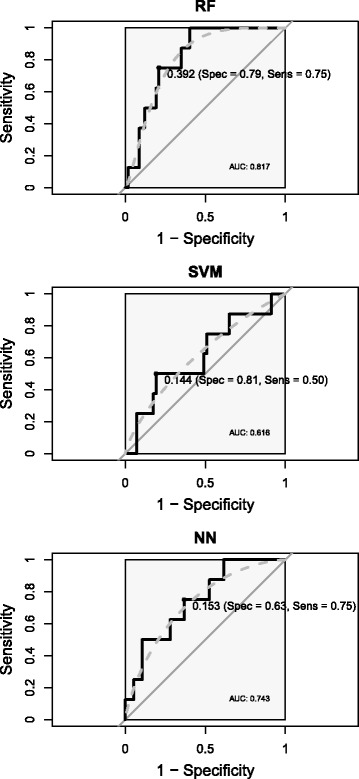
ROC curves. From top to bottom: ROC curves and AUC values computed on the evaluation patterns for the RF, SVM and NN models, respectively. The point in each curve that gives the best balance between sensitivity and specificity rates has been marked and annotated with the corresponding “alternative” threshold and efficacy values. Solid black box: *AUC = 1* reference area. Dashed gray line: smoothed ROC curve. Solid gray line: random guess

On the other hand, after model training using this down-sampled set of patterns, we used the ROC curve to determine alternative cutoffs for the probabilities predicted by the model. Using this ROC curve, an appropriate balance between sensitivity and specificity can be determined. Although several techniques do exist for determining a new cutoff, the more general approach is to find the point on the ROC curve that is closest (i.e., the shortest distance) to the perfect model (with 100% sensitivity and 100% specificity), which is associated with the upper left corner of the plot [4]. To determine this cutoff point without biasing the results obtained from the final testing dataset, an independent evaluation dataset was used (see above). In Fig. 3 the ROC curves obtained from the RF, SVM and NN classifiers (corresponding to the performance results in the last row of each model’s data in Table 1) on the evaluation dataset is shown together with the computed alternative cutoff. As it can be observed in the figure, the alternative cutoff gives a better balance between sensitivity and specificity. However, as it can be observed in Table 5, this better balance between sensibility and specificity is obtained at the expense of accuracy. For comparison purposes, in Table 5 those performance results (on the testing set) from the RF model of Table 1 are shown again (computed alternative cutoff: 0.392), together with the results for this same model but with the standard cutoff of 0.5.

**Table 5:** Performance rates for RF with two alternative ROC cutoffs

Feature set	Accuracy	Sensitivity	Specificity	F-measure	MCC
Alternative cutoff: 0.392					
Primary (52)	0.5969	0.8125	0.5664	0.3333	0.2500
Tertiary (24)	0.6357	0.8750	0.6018	0.3733	0.3155
Whole (76)	0.7597	0.8125	0.7522	0.4561	0.3998
mRMR (54)	0.7597	0.7500	0.7611	0.4364	0.3668
Standard cutoff: 0.5					
Primary (52)	0.8062	0.1875	0.8938	0.1935	0.0836
Tertiary (24)	0.7907	0.5625	0.8230	0.4000	0.3044
Whole (76)	0.8372	0.5625	0.8761	0.4615	0.3777
mRMR (54)	0.8372	0.6250	0.8673	0.4878	0.4105

#### Feature selection with the mRMR method

We used the minimum redundancy maximum relevance (mRMR) method [25] to rank the importance of the 76 features, based on the trade-off between the relevance to the output (oxidable) and the redundancy between the input characteristics. This method is based on the concept of *mutual information*. Given two variables, *x* and *y*, their mutual information can be defined as

$$I(x,y) = \int \int p(x, y) log \frac{p(x,y)}{p(x)p(y)} dx dy. $$

When the goal is to select N features from the whole feature set (*Ω*), according to their minimum redundancy (among them) and maximum relevance (with respect to the target or output, *o*), the first feature added to this set of selected characteristics, *Ω*_*s*_, is selected according to the concept of maximum relevancy. That is, the feature *f* with the highest *I*(*f*,*o*). The rest of features are selected in an incremental way: earlier selected features remain in the feature set *Ω*_*s*_. Suppose *m* features have been already selected, and we want to select an additional feature from the set *Ω*_*p*_=*Ω*−*Ω*_*s*_

The next characteristic *f*_*j*_∈*Ω*_*p*_ to be selected, i.e. to be included in *Ω*_*s*_, is the one that maximises the *mRMR* function, given by

$$max_{f_{j} \in \Omega_{p}}\left[ I\left(\, f_{j}, o\right) - \frac{1}{m}\sum_{f_{i} \in \Omega_{s}}I\left(\, f_{j}, f_{i}\right)\right] $$

To determine the final set of *N* selected features, an incremental approach was followed: for each number of selected characteristics *N*=2,…76, a random forest was trained (with down-sampled patterns from the training set), its ROC’s cut-off probability was established using the evaluation set (see “Methods” section) and, finally the AUC for the testing set was measured. Following this strategy, a final set of *N*=54 features was identified as the optimal (maximum AUC) feature set.

The final set of 54 features, in order of selection by the mRMR algorithm, is the following (see Sec. *Feature Extraction* for a description of the characteristics): SASA.pdb, NT_M, away.ptm, CT_Q, CT_F, NT_R, NT_D, Met2Y, CT_L, nF.pdb, NT_I, Met2S, dpx, CT_G, NT_C, nY.chain, CT_D, NT_W, sd.entropy, CT_E, CT_H, CT_M, closestAro.chain, NT_K, CT_V, CT_A, closer10A.pdb, CT_R, CT_N, NT_A, NT_P, NT_N, CT_C, NT_L, SASA.chain, NT_E, CT_P, NT_H, CT_T, NT_F, NT_V, CT_K, NT_G, NT_T, Bfactor, nW.pdb, entropy, NT_Q, CT_S, NT_Y, CT_I, NT_S, Fd.pdb and CT_Y.

## Abbreviations

AUC: Area under receiver operating characteristic curve
GI: Gini-index importance
KNN: K-nearest neighbors
MCC: Matthews correlation coefficient
MetO: Methionine sulfoxide
ML: Machine learning
MLP: Multilayer perceptron
mRMR: Maximum relevance minimum redundancy
Msr: Methionine sulfoxide reductase
NN: Neural network
OOB: Out-of-bag
PTM: Post-translational modification
RBF: Radial basis function
RF: Random forest
ROC: Receiver operating characteristic
ROS: Reactive species of oxygen
SASA: Solvent accessible surface area
SVM: Support vector machine
